# *BRCA1/2* mutation carriers vs the general breast cancer population (*N* = 799,986): 21-gene assay-based molecular characterization

**DOI:** 10.1007/s10549-024-07271-4

**Published:** 2024-04-03

**Authors:** Rinat Yerushalmi, Adi Pomerantz, Ron Lewin, Shani Paluch-Shimon, Lior Soussan-Gutman, Frederick L. Baehner, Hillary Voet, Avital Bareket-Samish, Inbal Kedar, Yael Goldberg, Tamar Peretz-Yablonski, Luna Kadouri

**Affiliations:** 1 Davidoff Cancer Center, Rabin Medical Center, 39 Jabotinski St, 49414 Petah Tikva, Israel; 2 Sackler Faculty of Medicine, Tel Aviv University, Tel Aviv, Israel; 3 Radiation Oncology Dept, Sheba Medical Center, Ramat Gan, Israel; 4 Sharett Institute of Oncology, Hadassah Hebrew University Hospital, Jerusalem, Israel; 5 Faculty of Medicine, Hebrew University, Jerusalem, Israel; 6 Oncotest, Rhenium, Modi’in, Israel; 7 Medical Department, Exact Sciences, Redwood City, CA USA; 8 Environmental Economics and Management, Hebrew University of Jerusalem, Rehovot, Israel; 9 BioInsight Ltd, Binyamina, Israel; 10 Rabin Medical Center, Raphael Recanati Genetic Institute, Petah Tikva, Israel

**Keywords:** 21-gene assay, BRCA, Breast cancer, Clinical outcomes, Pathogenic variant, Recurrence Score

## Abstract

**Purpose:**

We compared 21-gene recurrence score (RS) distribution and expression of the single-gene/gene groups within this assay between BC patients with pathogenic variants (PV) in *BRCA1/2* vs the general 21-gene-tested BC population.

**Methods:**

This retrospective study included consecutive 21-gene-tested female ER + HER2-negative BC patients with germline PVs in *BRCA1/2*. RS/gene expression data were compared to a previously described commercial use database (CDB, *N* = 799,986). Chi-square and 1-sample *t* test were used to compare RS distribution and single-gene/gene group scores between the study group and the CDB.

**Results:**

Study group patients (*N* = 81) were younger and their RS results were higher compared to the CDB (age: median [IQR], 56 [47–61.5] vs 60 [51–67] years; *p* < 0.001; proportion of patients with RS ≥ 26: 49.4% vs 16.4%, *p* < 0.001). Expression of 12/16 cancer genes in the assay and the ER, proliferation, and invasion gene group scores differed significantly between the study group and the CDB, all in a direction contributing to higher RS. The differences between the study group and the CDB were mostly retained, upon stratifying the patients by menopausal status.

**Conclusion:**

BC patients with PVs in *BRCA1/2* have higher RS results that stem from distinct gene expression profiles in the majority of genes in the 21-gene assay.

**Supplementary Information:**

The online version contains supplementary material available at 10.1007/s10549-024-07271-4.

## Introduction

The 21-gene Oncotype DX Breast Recurrence Score^®^ assay is used to guide adjuvant treatment in hormone receptor (HR) + HER2-negative early-stage breast cancer (BC) [1, 2]. The assay measures RNA expression of 16 cancer-related and 5 reference genes using quantitative real-time reverse transcriptase-polymerase chain reaction (qRT-PCR) on tumor tissue samples and calculates the Recurrence Score^®^ (RS; range: 0–100), which is a validated prognosticator and predictor of chemotherapy benefit [1–5]. The cancer-related genes include 4 linked to the estrogen-signaling pathway (*ESR1, PGR, BCL2,* and *SCUBE2*), 5 to proliferation/anti-apoptosis (*CCNB1, KI67, STK15, SURV,* and *MYBL2*), 2 to the HER2 pathway (*ERBB2* and *GRB7*), 2 to invasion (*STMY3* and *CTSL2)*, and 3 (*CD68, GSTM1,* and *BAG1*) to macrophage function, detoxification, and apoptosis, respectively. The levels of the cancer-related genes are normalized using the 5 reference genes [3]. The 21-gene assay report provides the RS result and single-gene scores for the estrogen receptor (*ESR1*), progesterone receptor (*PGR*), and *ERBB2*. The other 13 single-gene scores and gene group scores are not provided.

Germline pathogenic variants (PVs) in the *BRCA1/2* are associated with a higher risk of developing BC, which is more likely to have aggressive disease characteristics [6, 7]. The prevalence of PVs in *BRCA1/2* in BC patients varies between ethnic groups, with the highest prevalence among Ashkenazi Jews (8%) [8].

The 21-gene assay is offered to BC patients irrespective of *BRCA1/2* status. In fact, often, the *BRCA1/2* status comes to light after the 21-gene testing*.* Although the RS distribution in BC patients with PVs in *BRCA1/2* was shown to be shifted toward higher RS results [9–15], information on the molecular basis of this shift and the association with clinicopathological characteristics and clinical outcomes is lacking.

We compared RS distribution and expression of single-gene/gene groups between a study group which consisted of estrogen receptor (ER) + HER2-negative BC patients with PVs in *BRCA1/2* and the general 21-gene-tested BC patient population as reflected in a commercial use database (CDB) [16] and evaluated the associations between the RS result, single-gene/gene group expression, disease characteristics, and clinical outcomes in the study group.

## Materials and methods

### Study design

This retrospective cohort study included consecutive female patients with germline PVs in *BRCA1/2* and N0/N1mi/N1 ER + HER2-negative BC who underwent 21-gene testing through Clalit Health Services between 2004 and 2015 and received treatment at Rabin Medical Center (RMC) or Hadassah Medical Center (HMC). No exclusion criteria were applied. RS data and single-gene/gene group expression data from the study group were compared to those from the CDB which included 799,986 BC excisional samples [16].

The study was conducted in accordance with the Declaration of Helsinki. It was approved by the institutional review boards of RMC and HMC (approval #0043-14-RMC and #0227-20-HMO) and was granted a waiver for obtaining patient consent due to its retrospective design.

### Statistical considerations

Descriptive statistics were used to summarize clinicopathological characteristics and chemotherapy use in the study group. Chi-square and Wilcoxon signed-rank test were used to compare categorical and continuous parameters, respectively, between the study group and the CDB [16]. Fisher’s exact test was used to compare categorical parameters between patients with germline PVs in *BRCA1* vs *BRCA2*.

One sample *t* test was used to compare the expression of each of the 16 cancer genes and the gene group scores in the study group to the CDB, for all patients, by menopausal status (for CDB, age was used as a surrogate for menopausal status), age, and *BRCA*-mutated gene. For the comparison to the CDB, group scores were calculated as in Paik et al. [3] without correction for the HER2 and the proliferation group scores. Independent sample t test was used to compare gene expression and gene group scores (calculated as in Paik et al. [3] with the correction as described therein) between patient categories within the study group.

Within the study group, Fisher’s exact test was used to compare patients with and without distant recurrence, with respect to categorical patient/tumor parameters and treatments received. Logistic regression was used to determine the association between the gene group scores (calculated with correction) as continuous parameters and having a distant recurrence.

JMP^®^ Version 16 (SAS Institute Inc., Cary, NC) was used. All tests were 2-sided. *p* ≤ 0.05 was considered statistically significant.

## Results

### Patient characteristics

The study group included 81 female patients (all of whom were self-reported women), whereas the CDB included 799,986 BC excisional samples [16]. Baseline patient and tumor characteristics for the study group are presented in Table 1. Age at diagnosis was statistically significantly younger in the study group vs the CDB (median [IQR] 56 [47–61.5] vs 60 (51–67) years; *p* < 0.001). Also, in the study group, 29.6% were < 50 years at diagnosis vs 20.5% in the CDB (*p* = 0.030). The nodal status distribution in the study group and the CDB were similar: 80.2, 4.9, and 14.8% had N0, N1mi, and N1 disease, respectively, in the study group vs 84, 5, and 11%, respectively, in the CDB (*p* = 0.55). In the study group, more patients had PVs in *BRCA2* than *BRCA1* (59.3% vs. 39.5%). *BRCA* mutation information was unavailable for one patient.

**Table 1:** Baseline patient and tumor characteristics of the study group (*N* = 81)

Characteristics	Cases, No (%)
Median (interquartile range) age, years	56 (47–61.5)
Age category	
< 40 years	5 (6)
40–49 years	19 (23)
50–59 years	32 (40)
60–69 years	20 (25)
70–79 years	5 (6)
Menopausal status	
pre	28 (35)
Post	53 (65)
Nodal status	
N0	65 (80)
N1mi	4 (5)
N1	12 (15)
Median (interquartile range) tumor size in the greatest dimension, cm	1.5 (1.0–2.0)
Tumor size category	
≤ 1 cm	21 (26)
> 1—2 cm	43 (53)
> 2	16 (20)
Unknown	1 (1)
Tumor grade category	
Grade 1	5 (6)
Grade 2	36 (44)
Grade 3	33 (41)
Not applicable/Unknown^a^	7 (9)
*BRCA* mutation type	
* BRCA1*	32 (40)
* BRCA2*	48 (59)
Unknown	1 (1)

^a^3/7 (43%) of unknown tumor grade are invasive lobular carcinoma

Age at diagnosis was not statistically significantly different between patients with PVs in *BRCA1* vs *BRCA2* (median [IQR]: 52 (45.5–59) vs 57 (47–63) years, respectively; *p* = 0.17), whereas grade distribution did. In patients with PVs in *BRCA1*, grade information was available for 30 patients (11 [36.7%] with grade 1–2, 19 [63.3%] with grade 3). In patients with PVs in *BRCA2*, grade information was available for 43 (30 [69.8%] with grade 1–2, 13 [30.2%] with grade 3) (*p* = 0.005).

### RS results

**Fig. 1:**
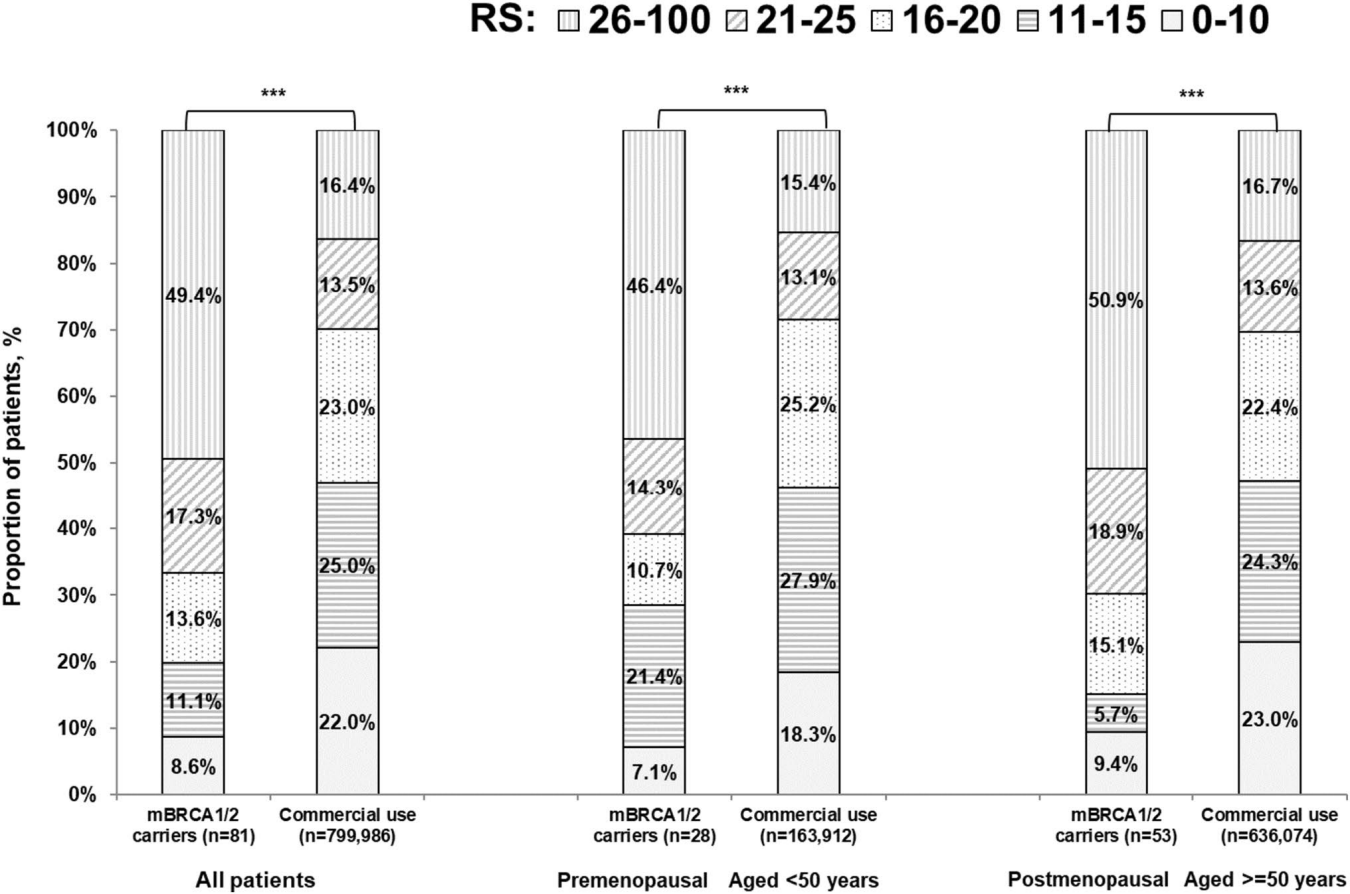
Distribution of RS results in the study group vs that in the CDB [16], overall and by menopausal status (for the CDB, age at diagnosis was used as a surrogate for menopausal status; < 50 vs ≥ 50 years). ***p < 0.001 (chi-square test)

The median RS result of the study group was statistically significantly higher than that of the CDB (25 [IQR, 18–35] vs 16 [IQR, 11–22]; *p* < 0.001). RS distribution also differed, with higher proportion of patients with RS 26–100 in the study group vs the CDB (49.4% vs 16.4%, *p* < 0.001) (Fig. 1).

RS results were also analyzed by *BRCA*-mutated gene (*BRCA1* vs *BRCA2*). The median (IQR) RS result for the 32 patients with PVs in *BRCA1* was 29 (18–37) vs 24 (16–31) for the 48 patients with PVs in *BRCA2* (*p* = 0.18). Both were statistically significantly different than the median RS result of the CDB (16 [IQR, 11–22]) (*p* < 0.001 each). Among patients with PVs in *BRCA1*, the number of patients in the RS 0–10, 11–15, 16–20, 21–25, and 26–100 categories was 2 (6.3%), 3 (9.4%), 6 (18.8%), 3 (9.4%), and 18 (56.3%), respectively, whereas among patients with PVs in *BRCA2*, the respective values were 5 (10.4%), 6 (12.5%), 5 (10.4%), 11 (22.9%), and 21 (43.8%). Both these RS distributions differed significantly from the corresponding distribution in the CDB with a shift toward the high-risk RS category regardless of the *BRCA*-mutated gene (*p* < 0.001, each) (Suppl. Figure 1).

The shift toward higher risk in patients with PVs in *BRCA1/2* was observed for premenopausal and postmenopausal patients separately (Fig. 1), as well as for younger and older patients separately (< 50, ≥ 50 years) (Suppl. Figure 2). Menopausal status information was not available for the CDB and a cut-off value of 50 years at diagnosis was used as a surrogate.

### Single-gene expression and gene group scores overall and by *BRCA*-mutated gene

The expression of 12 of the 16 cancer genes differed significantly between the study group and the CDB. In all, the directionality of the difference contributed to higher RS results (lower expression in study group patients vs the CDB in *PGR, SCUBE2*, *GSTM1,* and *BAG1;* higher expression in *CCNB1, KI67*, *STK1*5, *SURV, MYBL2, GRB7*, and *CTSL2*). Gene group scores for the ER, proliferation, and invasion gene groups, but not the HER2 gene group, differed significantly between the study group and the CDB, with the directionality of these differences contributing to higher RS results (lower ER gene group score and higher proliferation and invasion gene group scores in study group patients vs the CDB) (Table 2).

**Table 2:** Single-gene expression and gene group scores vs the commercial use database [16]: Overall and by *BRCA* mutation type

	All patients			Patients with PVs in *BRCA1*		Patients with PVs in *BRCA2*		
Gene	Patients with PVs in* BRCA1/2* mean (SD) *N* = 81	Commercial use database mean *N* = 799,986	*p*	Patients with PVs in* BRCA1* mean (SD) *n* = 32	*p-value* Patients with PVs in* BRCA1* vs the commercial use database	Patients with PVs in* BRCA2* mean (SD) *n* = 48	*p-value* Patients with PVs in* BRCA2* vs the commercial use database	*p-value* Patients with PVs in* BRCA1* vs patients with PVs in* BRCA2*
ER group								
* ESR1*	9.76 (1.38)	10.05	0.065	9.37 (1.54)	**0.018**	10.01 (1.22)	0.83	**0.041**
* PGR*	**6.25 (1.71)**	**7.29**	** < 0.001**	6.08 (1.75)	** < 0.001**	6.43 (1.64)	** < 0.001**	0.37
* BCL2*	8.56 (1.04)	8.52	0.75	8.32 (1.12)	0.32	8.69 (0.96)	0.23	0.12
* SCUBE2*	**8.21 (1.77)**	**8.83**	**0.0023**	7.91 (1.98)	**0.013**	8.38 (1.61)	0.054	0.25
Group score	**8.02 (1.07)**	**8.53**	** < 0.001**	7.75 (1.19)	** < 0.001**	8.20 (0.97)	**0.019**	0.073
Proliferation group								
* CCNB1*	**5.94 (0.52)**	**5.69**	** < 0.001**	6.00 (0.43)	** < 0.001**	5.89 (0.57)	**0.023**	0.34
* KI67*	**7.07 (0.82)**	**6.37**	** < 0.001**	7.15 (0.74)	** < 0.001**	7.00 (0.88)	** < 0.001**	0.42
* STK15*	**6.40 (0.76)**	**5.65**	** < 0.001**	6.45 (0.76)	** < 0.001**	6.34 (0.75)	** < 0.001**	0.54
* SURV*	**6.07 (1.13)**	**5.02**	** < 0.001**	6.18 (0.89)	** < 0.001**	5.97 (1.25)	** < 0.001**	0.40
* MYBL2*	**5.56 (0.96)**	**4.55**	** < 0.001**	5.83 (0.81)	** < 0.001**	5.37 (1.03)	** < 0.001**	**0.037**
Group score^a^	**6.21 (0.72)**	**5.46**	** < 0.001**	6.66 (0.27)/ 6.32 (0.59)	** < 0.001**	6.64 (0.24)/ 6.11 (0.79)	** < 0.001**	0.76
HER2 group								
* ERBB2*	9.06 (0.72)	9.16	0.21	8.92 (0.85)	0.12	9.15 (0.62)	0.92	0.16
* GRB7*	**6.84 (0.73)**	**6.68**	**0.049**	6.74 (0.79)	0.68	6.90 (0.69)	**0.031**	0.33
Group score^a^	7.07 (0.71)	6.93	0.091	8.02 (0.11)/ 6.96 (0.78)	0.84	8.03 (0.13)/ 7.13 (0.67)	**0.047**	0.74
Invasion group								
* STMY3*	9.84 (1.36)	10.04	0.18	9.74 (1.39)	0.22	9.95 (1.33)	0.64	0.49
* CTSL2*	**4.35 (0.89)**	**3.81**	** < 0.001**	4.35 (0.93)	**0.0026**	4.32 (0.83)	** < 0.001**	0.89
Group score	**7.10 (0.75)**	**6.92**	**0.043**	7.04 (0.68)	0.34	7.14 (0.81)	0.076	0.59
Individual								
* CD68*	**9.00 (0.55)**	**8.83**	**0.0070**	9.06 (0.58)	**0.035**	8.95 (0.54)	0.11	0.42
* GSTM1*	**6.90 (1.35)**	**7.84**	** < 0.001**	6.68 (1.56)	** < 0.001**	7.02 (1.18)	** < 0.001**	0.28
* BAG1*	**8.20 (0.61)**	**8.48**	** < 0.001**	8.07 (0.67)	**0.0014**	8.28 (0.57)	**0.018**	0.13

*PV* pathogenic variant

Bold entries designate statistical significance

^a^Gene group scores were calculated according to Paik et al. [3] with and without correction, respectively, as described therein. *mBRCA1* vs *mBRCA2* comparisons were performed with the corrected scores, and comparison to the commercial use database [16] was performed with the uncorrected gene group scores

Single-gene expression/gene group scores were compared between study group patients with PVs in *BRCA1* and *BRCA2* (Table 2). The only statistically significant difference between the *BRCA* mutation subgroups involved the *ESR1* gene, where the expression was higher in patients with PVs in *BRCA2* (mean [SD], 10.01 [1.22] vs 9.37 [1.54]; *p* = 0.041) and the *MYBL2* gene, where the expression was higher in patients with PVs in *BRCA1* (mean [SD], 5.83 [0.81] vs 5.37 [1.03]; *p* = 0.037).

Comparing study group patients with PVs in *BRCA1* to the CDB revealed statistically significant differences in single-gene expression in 12 of the 16 cancer genes and in 2 gene group scores, all in a direction that contributed to higher RS results in the study patients (lower expression in study group patients vs the CDB in *ESR1, PGR, SCUBE2*, *GSTM1, BAG1*, and the ER gene group score; higher expression in *CCNB1, KI67*, *STK1*5, *SURV, MYBL2, CTSL2,* and *CDK68*, and the proliferation gene group score). A similar analysis for patients with PVs in *BRCA2* revealed statistically significant differences in gene expression in 10 of the 16 cancer genes, and in 3 gene group scores, all in a direction that contributed to higher RS results in the study patients (lower expression in study group patients vs the CDB in *PGR*, *GSTM1, BAG1*, and the ER gene group score; higher expression in *CCNB1, KI67*, *STK1*5, *SURV, MYBL2, GRB7,* and *CTSL2,* and the *HER2* and the proliferation gene group scores) (Table 2).

### Single-gene/gene groups analyses by menopausal status and age

In the study group, the only statistically significant difference between pre- and postmenopausal patients was the expression of *PGR*, which was higher in premenopausal vs postmenopausal patients (mean [SD], 6.98 [1.45] vs 5.86 [1.72], *p* = 0.0046). Although menopausal status information was not available for the CDB, a similar finding was noted in the CDB upon using age as a surrogate (mean [SD] for CDB patients < 50 and ≥ 50 years: 7.77 [1.47] vs 7.16 [1.83], *p* < 0.001) (Table 3).

**Table 3:** Single-gene expression and gene group scores by menopausal status, in the study group vs the commercial use database [16]

	Premenopausal (Study Group) or < 50 years at Diagnosis (Commercial-use Database)			Postmenopausal (Study Group) or ≥ 50 years at Diagnosis (Commercial-use Database)		
Gene	Patients with PVs in* BRCA1/2* mean (SD) *N* = 28	Commercial use database mean *N* = 163,912	*p*	Patients with PVs in* BRCA1/2* mean (SD) *N* = 53	Commercial use database mean *N* = 636,074	*p*
ER group						
*ESR1*	9.38 (0.97)	9.28	0.61	9.96 (1.52)	10.24	0.069
*PGR*	**6.98 (1.45)**^**1**^	**7.77**	**0.0076**	**5.86 (1.72)**^**a**^	**7.16**	** < 0.001**
*BCL2*	8.74 (1.07)	8.53	0.33	8.46 (1.02)	8.52	0.70
*SCUBE2*	8.39 (1.73)	8.68	0.38	**8.12 (1.80)**	**8.87**	**0.0036**
Group score^b^	8.25 (0.81)	8.49	0.13	**7.90 (1.18)**	**8.54**	** < 0.001**
Proliferation group						
*CCNB1*	**5.98 (0.44)**	**5.70**	**0.0025**	**5.92 (0.56)**	**5.69**	**0.0050**
*KI67*	**7.09 (0.77)**	**6.41**	** < 0.001**	**7.06 (0.85)**	**6.36**	** < 0.001**
*STK15*	**6.49 (0.69)**	**5.64**	** < 0.001**	**6.35 (0.79)**	**5.65**	** < 0.001**
*SURV*	**6.14 (1.18)**	**5.02**	** < 0.001**	**6.04 (1.10)**	**5.02**	** < 0.001**
*MYBL2*	**5.64 (0.93)**	**4.66**	** < 0.001**	**5.52 (0.99)**	**4.52**	** < 0.001**
Group score^b^	**6.27 (0.70)**	**5.49**	** < 0.001**	6.18 (0.74)	**5.45**	** < 0.001**
HER2 group						
*ERBB2*	9.10 (0.88)	9.13	0.82	9.04 (0.63)	9.17	0.15
*GRB7*	6.86 (0.92)	6.71	0.39	6.84 (0.62)	6.68	0.065
Group score^b^	7.08 (0.90)	6.95	0**.**44	7.06 (0.60)	6.92	0.11
Invasion group						
*STMY3*	10.21 (1.24)	10.07	0.53	**9.65 (1.39)**	**10.04**	**0.045**
*CTSL2*	**4.29 (0.72)**	**3.67**	** < 0.001**	**4.39 (0.97)**	**3.84**	** < 0.001**
Group score^b^	**7.25 (0.73)**	**6.87**	**0.010**	7.02 (0.76)	6.94	0.47
Individual						
*CD68*	**8.95 (0.60)**	**8.67**	**0.019**	**9.03 (0.53)**	**8.87**	**0.038**
*GSTM1*	**7.05 (1.17)**	**7.72**	**0.0054**	**6.82 (1.44)**	**7.87**	** < 0.001**
*BAG1*	**8.20 (0.60)**	**8.50**	**0.013**	**8.19 (0.63)**	**8.48**	**0.0017**

*PV* pathogenic variant

Bold entries designate statistical significance

^a^*p* = 0.0046 for comparing *PGR* expression between pre- and postmenopausal patients (independent sample *t* test)

^b^Gene group scores were calculated as in Paik et al. [3] without correction

Single-gene expression/gene group scores analysis by menopausal status (using age as a surrogate for the CDB) demonstrated that the differences between the study group and the CDB observed for the entire cohort were mostly retained (except for *SCUBE2* in premenopausal patients, *GRB7* in both pre- and postmenopausal patients, the ER group score in premenopausal patients, and the invasion group score in postmenopausal patients). For the proliferation gene group, the differences in each single-gene and the gene group score remained highly significant in both pre- and postmenopausal patients (Table 3).

Analysis of single-gene/gene group score by age within the study group and in comparison to the corresponding age groups in the CDB, yielded similar results to those found when the study group was stratified by menopausal status (Suppl. Table 1).

### Single-gene expression by other patient/disease characteristics

Single-gene expression was similar in N0 patients vs N1mi/N1 patients, except for differences in the *BAG1* gene whose expression was higher in N1mi/N1 patients (mean [SD], 8.48 [0.65] vs 8.13 [0.59], *p* = 0.037), and the *PGR* gene whose expression was also higher in N1mi/N1 patients (mean [SD], 7.10 [1.33] vs 6.04 [1.73], *p* = 0.025; Suppl. Table 2). In both genes, the directionality of the differences contributed to higher RS results in patients with N0 disease.

Expression of 10 genes differed significantly between patients with grade 1–2 tumors vs patients with grade 3 tumors. These included all the genes in the ER group whose expression was higher in grade 1–2 vs grade 3 tumors. Consequently, the ER gene group score was also significantly higher in grade 1–2 tumors. Additionally, the *GSTM1*, *BAG1,* and *ERBB2* genes had significantly higher expression in grade 1–2 tumors and 3 genes in the proliferation group (*STK15, SURV,* and *MYBL2*) had higher expression in grade 3 tumors. Except for the *ERBB2* gene, the directionality of the differences contributed to higher RS results in patients with grade 3 tumors (Suppl. Table 2).

### Treatments and clinical outcomes in the study group

Overall, 37 (45.7%) patients in the study group received adjuvant chemotherapy. Of the 32 patients with PVs in *BRCA1*, 18 (56.3%) received chemotherapy including 1/14 (7.1%) with RS 0–25, and 17/18 (94.4%) with RS 26–100 (for one patient in the RS 26–100 group, treatment information was unavailable). Of the 48 patients with PVs in *BRCA2*, 19 (39.6%) received chemotherapy including 5/27 (18.5%) with RS 0–25 and 14/21 (66.7%) with RS 26–100.

With a median follow-up of 8.2 (IQR, 5.6–9.7) years from diagnosis, one patient with PVs in *BRCA2* experienced BC recurrence in the same breast (3.8 years after the initial diagnosis), three had contralateral BC (1.3–6.5 years after their initial BC diagnosis), and nine experienced distant recurrence (of whom 1 had also the aforementioned recurrence in the same breast). Of these nine patients, 1 had PVs in *BRCA1* and 8 in *BRCA2*. The median RS result of these 9 patients was 25 (range, 16–41) and 4 received adjuvant chemotherapy. The distant recurrence occurred 31–130 months from the BC diagnosis (Table 4).

**Table 4:** Baseline patient/disease characteristics, treatments, and clinical outcomes of distant recurrence cases in the study group

#	Type of *BRCA* mutation	RS	Age, years	Menopausal Status	Tumor size, cm	N	Grade	Type of primary surgery	ALND	Adjuvant radiation	Type of hormonal therapy	Type of adjuvant chemotherapy	Location of metastases	DRFS, months
1	*BRCA1*	18	36	pre	1.0	N1	2	Mastectomy	Yes	No	Tamoxifen; AI	None	Visceral	58
2	*BRCA2*	16	42	pre	2.0	N1	2	Lumpectomy	NA	Yes	Tamoxifen	None	Other	31
3	*BRCA2*	21	57	post	1.6	N0	2	Mastectomy	No	No	NA	None	Bones	NA
4	*BRCA2*	23	31	pre	2.5	N0	2	Lumpectomy	NA	Yes	Tamoxifen	CMF	Bones^a^	46
5	*BRCA2*	25	72	post	1.5	N1mi	2	Lumpectomy	No	Yes	Tamoxifen; AI	None	Visceral	90
6	*BRCA2*	28	59	post	1.7	N0	NA	Lumpectomy	No	Yes	Tamoxifen; AI	None	Bones and visceral	75
7	*BRCA2*	31	63	post	2.3	N0	3	Lumpectomy	No	Yes	AI	Carboplatin plus paclitaxel	Visceral	99
8	*BRCA2*	31	72	post	1.5	N0	3	Lumpectomy	No	Yes	AI	AC	Bones	130
9	*BRCA2*	41	47	pre	2.0	N0	3	NA	No	No	Tamoxifen	AC	Visceral	107

*AC* Adriamycin and cyclophosphamide; *AI* aromatase inhibitor; *ALND* axillary lymph node dissection; *CMF* cyclophosphamide, methotrexate, and fluorouracil; *DRFS* distant recurrence-free survival; *NA* not available; *RS* Recurrence Score

^a^This patient experienced recurrence in the same breast at the same time the bone metastases were diagnosed

No statistically significant differences between the nine patients with distant recurrence and the 72 non-recurring patients were observed in terms of patient/disease characteristics (age, menopausal status, tumor grade, nodal status, RS category, and *BRCA* mutation type) and treatment with chemotherapy (Suppl. Table 3). A trend toward significance was observed with respect to *BRCA* mutation type, with recurrences among 1/32 [3.1%] patients with PVs in *BRCA1* vs 8/48 [16.7%] of patients with PVs in *BRCA2*, *p* = 0.078). The proliferation and invasion gene group scores were significantly associated with the odds of having distant recurrence (proliferation group score: odds ratio [OR], 23.60 [95% CI, 1.4–396.9], *p* = 0.028; invasion group score: OR, 5.12 [95% CI, 1.13–23.12], *p* = 0.034). The ER and HER2 gene group scores were not associated with distant recurrence (Suppl. Table 4).

## Discussion

This study, which compared RS results, single-gene expression of the 16 cancer genes within the 21-gene assay, and gene group scores between a cohort of ER + BC patients with PVs in *BRCA1/2* (*N* = 81) and all 21-gene-tested BC patients (*N* = 799,986) demonstrated that those with PVs in *BRCA1/2* had higher RS results that could not be attributed to menopausal status or age at diagnosis and stemmed from a distinct gene expression profile of the majority of these 16 cancer genes. Our findings are consistent with prior studies showing higher RS results in patients with PVs in *BRCA1/2* [9–15]; however, this is the first study to compare the single-gene expression of the cancer genes within the assay between patients with PVs in *BRCA1/2* and the general 21-gene-tested populations.

This study was also the first to explore single-gene differences between patients with PVs in *BRCA1* vs *BRCA2*. The RS result in patients with PVs in *BRCA1* was numerically higher than in *BRCA2* patients, although the difference was not statistically significantly different, which is consistent with prior studies [9–11, 14]. *ESR1* gene expression was significantly lower in patients with PVs in *BRCA1* vs *BRCA2*, which is consistent with a study comparing 20 patients with PVs in *BRCA1*, 38 patients with PVs in *BRCA2*, and 1020 controls, where the ER index in those with PVs in *BRCA1* but not *BRCA2* was statistically significantly lower than that in the controls [9]. This known association between *BRCA1* mutation subtype and lower ER expression, may have contributed to the higher chemotherapy use observed in those with PVs in *BRCA1* vs *BRCA2* (56.3% vs 39.6%).

Analysis of single-gene expression in the study patients by grade revealed significant differences in the expression of 10 genes, all except 1 (*ERBB2*) in a directionality contributing to higher RS results in grade 3 tumors, which is consistent with the known relationship between RS result and grade [17]. In the study group, single-gene expression was overall similar between pre- and postmenopausal patients as well as between N0 and N1mi/N1 patients. The only gene with differential expression by menopausal status was *PGR*, whose expression was higher in pre- compared to the postmenopausal study patients. The same result was demonstrated in the CDB (using age as a surrogate), suggesting that the differential *PGR* expression by age/menopausal status may be unrelated to *BRCA* status. Interestingly, in the study cohort, N1mi/N1 patients had higher *PGR* expression compared to N0 patients. Since *PGR* expression data by nodal status was unavailable for the study cohort, this observation warrants further investigation, particularly as positive nodes are associated with higher clinical risk in BC, whereas high *PGR* levels are associated with lower clinical risk [18, 19].

Our findings demonstrate that patients with PVs in *BRCA1/2* are likely to have higher RS results and therefore suggest that the high-risk RS group is enriched with patients who have PVs in *BRCA1/2*. This observation should be considered when discussing the RS results with patients. The observed similarity in gene expression in study group patients regardless of age/menopausal status emphasizes the unique gene pattern of this population. There are no data to indicate that the 21-gene assay should be used differently in patients with PVs in *BRCA1/2*. Our study demonstrated a statistically significant association between the proliferation and invasion gene group scores and having a distant recurrence. There was no correlation with the ER group. Further studies with larger cohorts of patients with PVs in *BRCA1/2* are warranted to better define the RS threshold and chemotherapy benefit in these patients.

The strengths of our study include its representation of real-world clinical practice, a long follow-up, and extensive clinical data on each patient in the study group. Another strength involves the robust control dataset of nearly 800,000 samples from various countries worldwide. Notably, although these countries may differ in the assay eligibility criteria, the size of the dataset mitigates against a substantial selection bias effect. Moreover, the consistency in our findings between younger and older patients further supports the absence of such a bias effect. Our study is limited by the sample size of the study group and the small number of events. Also, the CDB includes all 21-gene-tested patients regardless of *BRCA1/2* status; however, the proportion of patients with PVs in *BRCA1/2* in the CDB is negligible due to the very low prevalence of such mutations [7, 8, 20].

In conclusion, patients with ER + HER2-negative early BC and PVs in *BRCA1/2* were characterized by higher RS results that stemmed from a distinct gene expression profile of most genes in the 21-gene assay. Further study is required to explore whether these patients should have a distinct model or RS threshold for considering chemotherapy use.

### Supplementary Information

Below is the link to the electronic supplementary material.

Supplementary file1 (DOC 290 KB)

## Data Availability

Data generated during and/or analyzed during the current study are available from the corresponding author on reasonable request.

## References

[1] Ahmed S, Pati S, Le D, Haider K, Iqbal N (2020). The prognostic and predictive role of 21-gene recurrence scores in hormone receptor-positive early-stage breast cancer. J Surg Oncol.

[2] Markopoulos C, Hyams DM, Gomez HL, Harries M, Nakamura S, Traina T, Katz A (2020). Multigene assays in early breast cancer: insights from recent phase 3 studies. Eur J Surg Oncol.

[3] Paik S, Shak S, Tang G, Kim C, Baker J, Cronin M, Baehner FL, Walker MG, Watson D, Park T, Hiller W, Fisher ER, Wickerham DL, Bryant J, Wolmark N (2004). A multigene assay to predict recurrence of tamoxifen-treated, node-negative breast cancer. N Engl J Med.

[4] Sparano JA, Gray RJ, Makower DF, Pritchard KI, Albain KS, Hayes DF, Geyer CE, Dees EC, Goetz MP, Olson JA, Lively T, Badve SS, Saphner TJ, Wagner LI, Whelan TJ, Ellis MJ, Paik S, Wood WC, Ravdin PM, Keane MM, Gomez Moreno HL, Reddy PS, Goggins TF, Mayer IA, Brufsky AM, Toppmeyer DL, Kaklamani VG, Berenberg JL, Abrams J, Sledge GW (2018). Adjuvant chemotherapy guided by a 21-gene expression assay in breast cancer. N Engl J Med.

[5] Kalinsky K, Barlow WE, Gralow JR, Meric-Bernstam F, Albain KS, Hayes DF, Lin NU, Perez EA, Goldstein LJ, Chia SKL, Dhesy-Thind S, Rastogi P, Alba E, Delaloge S, Martin M, Kelly CM, Ruiz-Borrego M, Gil-Gil M, Arce-Salinas CH, Brain EGC, Lee ES, Pierga JY, Bermejo B, Ramos-Vazquez M, Jung KH, Ferrero JM, Schott AF, Shak S, Sharma P, Lew DL, Miao J, Tripathy D, Pusztai L, Hortobagyi GN (2021). 21-gene assay to inform chemotherapy benefit in node-positive breast cancer. N Engl J Med.

[6] Paul A, Paul S (2014). The breast cancer susceptibility genes (BRCA) in breast and ovarian cancers. Front Biosci (Landmark Ed).

[7] Atchley DP, Albarracin CT, Lopez A, Valero V, Amos CI, Gonzalez-Angulo AM, Hortobagyi GN, Arun BK (2008). Clinical and pathologic characteristics of patients with BRCA-positive and BRCA-negative breast cancer. J Clin Oncol.

[8] John EM (2007). Prevalence of pathogenic BRCA1 mutation carriers in 5 US racial/ethnic groups. JAMA.

[9] Lewin R, Sulkes A, Shochat T, Tsoref D, Rizel S, Liebermann N, Hendler D, Neiman V, Ben-Aharon I, Friedman E, Paluch-Shimon S, Margel D, Kedar I, Yerushalmi R (2016). Oncotype-DX recurrence score distribution in breast cancer patients with BRCA1/2 mutations. Breast Cancer Res Treat.

[10] Halpern N, Sonnenblick A, Uziely B, Divinsky L, Goldberg Y, Hamburger T, Peretz T, Kadouri L (2017). Oncotype Dx recurrence score among BRCA1/2 germline mutation carriers with hormone receptors positive breast cancer. Int J Cancer.

[11] Shah PD, Patil S, Dickler MN, Offit K, Hudis CA, Robson ME (2016). Twenty-one-gene recurrence score assay in BRCA-associated versus sporadic breast cancers: differences based on germline mutation status. Cancer.

[12] Blanter J, Zimmerman B, Tharakan S, Ru M, Cascetta K, Tiersten A (2020). BRCA mutation association with recurrence score and discordance in a large Oncotype database. Oncology.

[13] Layman RM, Lin H, Gutierrez Barrera AM, Karuturi MS, Yam C, Arun BK (2022). Clinical outcomes and Oncotype DX breast recurrence score(R) in early-stage BRCA-associated hormone receptor-positive breast cancer. Cancer Med.

[14] Casasanta N, Kipnis ST, Linville L, Lipinski S, Knoedler A, Marino A, McHenry A, Biagi T, Stark E, Amdur R, Denduluri N, Rodriguez P, Isaacs C, Kaltman R (2020). Relationship between hereditary cancer syndromes and Oncotype DX recurrence score. Clin Breast Cancer.

[15] Davey MG, Richard V, Lowery AJ, Kerin MJ (2021). OncotypeDX(c) recurrence score in BRCA mutation carriers: a systematic review and meta-analysis. Eur J Cancer.

[16] Jakubowski DM, Bailey H, Abran J, Blacklock A, Ciau N, Mies C, Tan V, Young R, Lau A, Baehner FL (2020). Molecular characterization of breast cancer needle core biopsy specimens by the 21-gene breast recurrence score test. J Surg Oncol.

[17] Singh K, He X, Kalife ET, Ehdaivand S, Wang Y, Sung CJ (2018). Relationship of histologic grade and histologic subtype with Oncotype Dx recurrence score; retrospective review of 863 breast cancer Oncotype Dx results. Breast Cancer Res Treat.

[18] Diana A, Carlino F, Buono G, Antoniol G, Famiglietti V, De Angelis C, Carrano S, Piccolo A, De Vita F, Ciardiello F, Daniele B, Arpino G, Orditura M (2022). Prognostic relevance of progesterone receptor levels in early luminal-like HER2 negative breast cancer subtypes: a retrospective analysis. Front Oncol.

[19] Ono M, Tsuda H, Yoshida M, Shimizu C, Kinoshita T, Tamura K (2017). Prognostic significance of progesterone receptor expression in estrogen-receptor positive, HER2-negative, node-negative invasive breast cancer with a low Ki-67 labeling index. Clin Breast Cancer.

[20] Li J, Wen WX, Eklund M, Kvist A, Eriksson M, Christensen HN, Torstensson A, Bajalica-Lagercrantz S, Dunning AM, Decker B, Allen J, Luccarini C, Pooley K, Simard J, Dorling L, Easton DF, Teo SH, Hall P, Borg Å, Grönberg H, Czene K (2019). Prevalence of BRCA1 and BRCA2 pathogenic variants in a large, unselected breast cancer cohort. Int J Cancer.

