# Supplementary material for: BRCA1/2 mutation carriers vs the general breast cancer population (N = 799,986): 21-gene assay-based molecular characterization
Source: Breast Cancer Res Treat. 2024 Apr 3;206(1):67–76. doi: 10.1007/s10549-024-07271-4 (PMC11182787; doi:10.1007/s10549-024-07271-4)
Supplement: Supplementary file 1 — Supplementary file1 (DOC 290 KB) [file 10549_2024_7271_MOESM1_ESM.doc]

**SUPPLEMENTARY MATERIALS**

**Supplementary Table 1** Single gene expression and gene group scores in the study group vs the commercial use database [1] by age category

|  | **All Patients** | | | **<50 years at Diagnosis** | | | **≥50 years at Diagnosis** | | |
| --- | --- | --- | --- | --- | --- | --- | --- | --- | --- |
| **Gene** | **Patients with PVs in *BRCA1/2***  **mean (SD)**  ***N* = 81** | **Commercial use database**  **mean**  ***N* = 799,986** | ***p*** | **Patients with PVs in *BRCA1/2***  **mean (SD)**  ***N* = 24** | **Commercial use database**  **mean**  ***N* = 163,912** | ***p*** | **Patients with PVs in *BRCA1/2***  **mean (SD)**  ***N* = 57** | **Commercial use database**  **mean**  ***N* = 636,074** | ***p*** |
| **ER group** |  |  |  |  |  |  |  |  |  |
| *ESR1* | 9.76 (1.38) | 10.05 | 0.065 | 9.55 (1.05) | 9.28 | 0.22 | **9.85 (1.49)** | **10.24** | **0.050** |
| ***PGR*** | **6.25 (1.71)** | **7.29** | **<0.001** | **7.18 (1.35)^a^** | **7.77** | **0.0042** | **5.86 (1.70)^a^** | **7.16** | **<0.001** |
| *BCL2* | 8.56 (1.04) | 8.52 | 0.75 | 8.73 (1.04) | 8.53 | 0.36 | 8.49 (1.04) | 8.52 | 0.82 |
| ***SCUBE2*** | **8.21 (1.77)** | **8.83** | **0.0023** | 8.50 (1.58) | 8.68 | 0.57 | **8.09 (1.85)** | **8.87** | **0.0023** |
| **Group score^b^** | **8.02 (1.07)** | **8.53** | **<0.001** | 8.37(0.71) | 8.49 | 0.42 | 7.87 (1.16) | 8.54 | **<0.001** |
| **Proliferation group** |  |  |  |  |  |  |  |  |  |
| ***CCNB1*** | **5.94 (0.52)** | **5.69** | **<0.001** | **6.02 (0.53)** | **5.70** | **0.0061** | **5.90 (0.51)** | **5.69** | **0.0033** |
| ***KI67*** | **7.07 (0.82)** | **6.37** | **<0.001** | **7.05 (0.95)** | **6.41** | **0.0031** | **7.08 (0.77)** | **6.36** | **<0.001** |
| ***STK15*** | **6.40 (0.76)** | **5.65** | **<0.001** | **6.48 (0.78)** | **5.64** | **<0.001** | **6.36 (0.75)** | **5.65** | **<0.001** |
| ***SURV*** | **6.07 (1.13)** | **5.02** | **<0.001** | **6.10 (1.43)** | **5.02** | **0.0012** | **6.06 (0.99)** | **5.02** | **<0.001** |
| ***MYBL2*** | **5.56 (0.96)** | **4.55** | **<0.001** | **5.65 (1.18)** | **4.66** | **<0.001** | **5.52 (0.87)** | **4.52** | **<0.001** |
| **Group score^b^** | **6.21 (0.72)** | **5.46** | **<0.001** | 6.26 (0.90) | **5.49** | **<0.001** | **6.19 (0.64)** | **5.45** | **<0.001** |
| **HER2 group** |  |  |  |  |  |  |  |  |  |
| *ERBB2* | 9.06 (0.72) | 9.16 | 0.21 | 9.10 (0.83) | 9.13 | 0.82 | 9.04 (0.67) | 9.17 | 0.17 |
| ***GRB7*** | **6.84 (0.73)** | **6.68** | **0.049** | 6.84 (0.81) | 6.71 | 0.43 | 6.85 (0.70) | 6.68 | 0.073 |
| **Group score^b^** | 7.07 (0.71) | 6.93 | 0.091 | 7.07 (0.80) | 6.95 | 0.49 | 7.07 (0.68) | 6.92 | 0.12 |
| **Invasion group** |  |  |  |  |  |  |  |  |  |
| *STMY3* | 9.84 (1.36) | 10.04 | 0.18 | 9.95 (1.62) | 10.07 | 0.73 | 9.80 (1.25) | 10.04 | 0.15 |
| ***CTSL2*** | **4.35 (0.89)** | **3.81** | **<0.001** | **4.30 (0.71)** | **3.67** | **<0.001** | **4.37 (0.96)** | **3.84** | **<0.001** |
| **Group score^b^** | **7.10 (0.75)** | **6.92** | **0.043** | 7.13 (0.90) | 6.87 | 0.18 | 7.09 (0.69) | 6.94 | 0.12 |
| **Individual** |  |  |  |  |  |  |  |  |  |
| ***CD68*** | **9.00 (0.55)** | **8.83** | **0.0070** | 8.85 (0.51) | 8.67 | 0.10 | **9.06 (0.56)** | **8.87** | **0.012** |
| ***GSTM1*** | **6.90 (1.35)** | **7.84** | **<0.001** | **6.95 (1.12)** | **7.72** | **0.0028** | **6.88 (1.44)** | **7.87** | **<0.001** |
| ***BAG1*** | **8.20 (0.61)** | **8.48** | **<0.001** | 8.40 (0.60) | 8.50 | 0.40 | **8.11 (0.60)** | **8.48** | **<0.001** |

*PV* pathogenic variant.

Note: Bold entries designate statistical significance (one-sample t-test, *p* ≤0.05).

^a^ *p*=0.0011 for comparing *PGR* expression between patients <50 years and ≥50 years at diagnosis (independent sample t-test).

^b^ Gene group scores were calculated as in Paik et al^2^ without correction.

**Supplementary Table 2** Single gene expression and gene group scores in the study group by nodal status and grade

| **Gene** | **N0 patients**  **mean (SD)**  ***N* = 65** | **N1mi/N1 patients**  **mean (SD)**  ***N* = 16** | ***p*** | **Grade 1-2**  **patients**  **mean (SD)**  ***N* = 41^a^** | **Grade 3**  **patients**  **mean (SD)**  ***N* = 33^a^** | ***p*** |
| --- | --- | --- | --- | --- | --- | --- |
| **ER group** |  |  |  |  |  |  |
| ***ESR1*** | 9.78 (1.46) | 9.69 (1.00) | 0.81 | **10.15 (1.08)** | **9.29 (1.54)** | **0.0063** |
| ***PGR*** | **6.04 (1.73)** | **7.10 (1.33)** | **0.025** | **6.61 (1.57)** | **5.75 (1.76)** | **0.031** |
| ***BCL2*** | 8.54 (1.09) | 8.63 (0.84) | 0.78 | **8.97 (0.79)** | **8.20 (1.19)** | **0.0015** |
| ***SCUBE2*** | 8.08 (1.80) | 8.74 (1.60) | 0.18 | **8.75 (1.77)** | **7.42 (1.53)** | **0.0011** |
| **Group score^b^** | 7.92 (1.11) | 8.41 (0.83) | 0.10 | **8.44 (0.85)** | **7.49 (1.11)** | **<0.001** |
| **Proliferation group** |  |  |  |  |  |  |
| ***CCNB1*** | 5.93 (0.52) | 5.96 (0.53) | 0.82 | 5.89 (0.54) | 6.03 (0.44) | 0.20 |
| ***KI67*** | 7.03 (0.84) | 7.26 (0.74) | 0.32 | 7.00 (0.87) | 7.23 (0.72) | 0.23 |
| ***STK15*** | 6.40 (0.79) | 6.41 (0.63) | 0.96 | **6.26 (0.84)** | **6.62 (0.65)** | **0.046** |
| ***SURV*** | 6.08 (1.16) | 6.06 (0.99) | 0.96 | **5.83 (1.23)** | **6.50 (0.87)** | **0.010** |
| ***MYBL2*** | 5.53 (0.99) | 5.71 (0.89) | 0.51 | **5.28 (1.00)** | **6.05 (0.68)** | **<0.001** |
| **Group score^b^** | 6.66 (0.26) | 6.64 (0.24) | 0.85 | 6.64 (0.25) | 6.71 (0.28) | 0.23 |
| **HER2 group** |  |  |  |  |  |  |
| ***ERBB2*** | 9.05 (0.74) | 9.11 (0.67) | 0.77 | **9.20 (0.66)** | **8.84 (0.73)** | **0.026** |
| ***GRB7*** | 6.86 (0.76) | 6.77 (0.64) | 0.65 | 6.90 (0.77) | 6.79 (0.71) | 0.55 |
| **Group score^b^** | 8.03 (0.13) | 8.00 (0.00) | 0.34 | 8.03 (0.14) | 8.02 (0.11) | 0.69 |
| **Invasion group** |  |  |  |  |  |  |
| ***STMY3*** | 9.79 (1.39) | 10.06 (1.25) | 0.49 | 10.00 (1.35) | 9.81 (1.29) | 0.53 |
| ***CTSL2*** | 4.34 (0.86) | 4.41 (1.00) | 0.76 | 4.26 (0.83) | 4.49 (0.97) | 0.28 |
| **Group score^b^** | 7.06 (0.78) | 7.23 (0.61) | 0.42 | 7.13 (0.74) | 7.15 (0.69 | 0.92 |
| **Individual** |  |  |  |  |  |  |
| ***CD68*** | 9.02 (0.55) | 8.92 (0.58) | 0.52 | 8.97 (0.56) | 9.06 (0.57) | 0.48 |
| ***GSTM1*** | 6.76 (1.33) | 7.48 (1.32) | 0.055 | **7.33 (1.15)** | **6.27 (1.29)** | **<0.001** |
| ***BAG1*** | **8.13 (0.59)** | **8.48 (0.65)** | **0.037** | **8.45 (0.51)** | **7.92 (0.64)** | **<0.001** |

Note: Bold entries designate statistical significance (independent sample t-test, *p* ≤0.05) between the different subgroups within the study group (N0 vs N1mi/N1 and Grade 1-2 vs Grade 3).

^a^ Grade information was unavailable for 7 patients.

^b^ Gene group scores were calculated as in Paik et al [2] with correction as described therein.

**Supplementary Table 3** Baseline disease characteristics, treatments, and clinical outcomes of patients with distant recurrence vs non-recurring patients in the study group

|  | **Distant Recurrence** | |  |
| --- | --- | --- | --- |
|  | **Yes** | **No** | ***p*** |
|  | **No. (%)** | **No. (%)** |
| **Age group** |  |  |  |
| <50 (*n* = 24) | 4 (17) | 20 (83) | 0.44 |
| ≥50 (*n* = 57) | 5 (9) | 52 (91) |
| All (*n* = 81) | 9 (11) | 72 (89) |  |
| **Menopausal status** |  |  |  |
| pre (*n* = 28) | 4 (14) | 24 (86) | 0.71 |
| post (*n* = 53) | 5 (9) | 48 (91) |
| All (*n* = 81) | 9 (11) | 72 (89) |  |
| **Tumor grade** |  |  |  |
| 1+2 (*n* = 41) | 5 (12) | 36 (88) | 0.73 |
| 3 (*n* = 33) | 3 (9) | 30 (91) |
| All (*n* = 74) | 8 (11) | 66 (89) |  |
| **Nodal status** |  |  |  |
| N0 (*n* = 65) | 6 (9) | 59 (91) | 0.37 |
| N1mi/N1 (*n* = 16) | 3 (19) | 13 (81) |
| All (*n* = 81) | 9 (11) | 72 (89) |  |
| **Type of *BRCA* mutation** |  |  |  |
| *BRCA1* (*n* = 32) | 1 (3) | 31 (97) | 0.078 |
| *BRCA2* (*n* = 48) | 8 (17) | 40 (83) |  |
| All (*n* = 80) | 9 (11) | 71 (89) |  |
| **RS category** |  |  |  |
| 0-25 (*n* = 41) | 5 (12) | 36 (88) | >0.99 |
| 26-100 (*n* = 40) | 4 (10) | 36 (90) |
| All (*n* = 81) | 9 (11) | 72 (89) |  |
| **Chemotherapy** |  |  |  |
| Yes (*n* =37) | 4 (11) | 33 (89) | 0.88 |
| No (*n* =42) | 5 (12) | 37 (88) |
| All (*n* =79) | 9 (11) | 70 (89) |  |

*RS* Recurrence Score.

**Supplementary Table** **4** Logistic regression model for the association between gene group scores and distant recurrence in the study group

| **Gene group** | **Odds ratio** | **95% CI** | ***p*** |
| --- | --- | --- | --- |
| ER group | 2.69 | (0.95-7.68) | 0.064 |
| **Proliferation group** | **23.604** | **(1.40-396.85)** | **0.028** |
| HER2 group | 0.00 | (0.00->10^7^) | 0.064 |
| **Invasion group** | **5.12** | **(1.13-23.12)** | **0.034** |

Note: Bold entries designate statistical significance (*p*≤0.05)

**Supplementary Fig. 1** Distribution of RS results in the study group vs the commercial use database [1] overall and by *BRCA* mutated gene (*BRCA1* vs *BRCA2*). ****p*<0.001 (chi-square test). *PV* pathogenic variant, *RS* Recurrence Score. *BRCA* mutation information was unavailable for one patient.

**
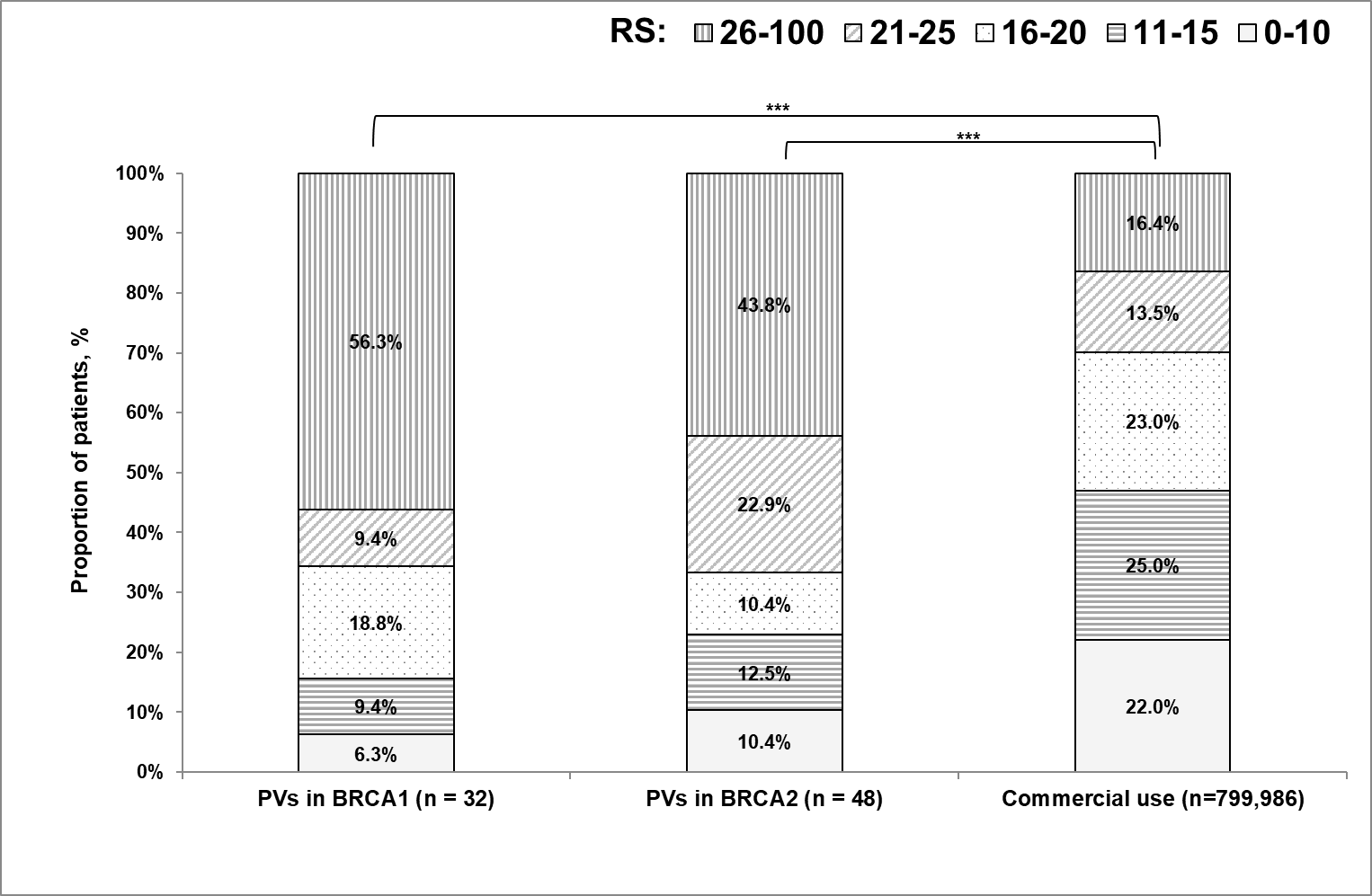
**

**Supplementary Fig. 2** Distribution of RS results in the study group vs the commercial use database [1] overall and by age group (<50 vs ≥50 years). * *p*<0.05; ****p*<0.001 (chi-square test). *RS* Recurrence Score.

**
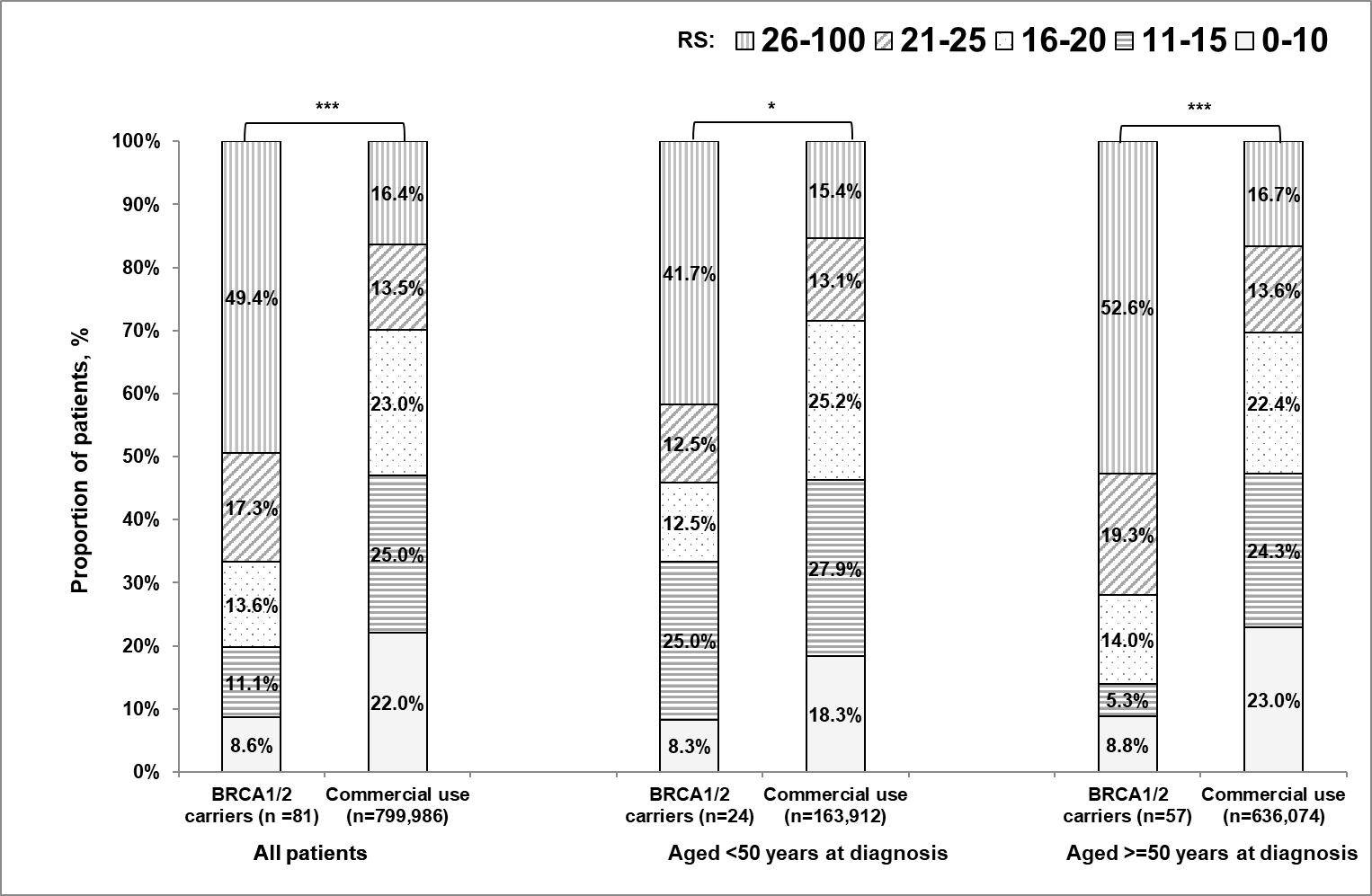
**

**References**

1. Jakubowski DM, Bailey H, Abran J, Blacklock A, Ciau N, Mies C, Tan V, Young R, Lau A, Baehner FL (2020) Molecular characterization of breast cancer needle core biopsy specimens by the 21-gene Breast Recurrence Score test. J Surg Oncol 122(4):611-618. https://doi.org/10.1002/jso.26050
2. Paik S, Shak S, Tang G, Kim C, Baker J, Cronin M, Baehner FL, Walker MG, Watson D, Park T, Hiller W, Fisher ER, Wickerham DL, Bryant J, Wolmark N (2004) A multigene assay to predict recurrence of tamoxifen-treated, node-negative breast cancer. N Engl J Med 351(27):2817-2826. https:/doi.org/10.1056/NEJMoa041588
